# A primary care database study of asthma among patients with and without opioid use disorders

**DOI:** 10.1038/s41533-020-0174-2

**Published:** 2020-04-20

**Authors:** Phillip Oliver, Joe Hulin, Caroline Mitchell

**Affiliations:** 1 Academic Unit of Primary Medical Care, University of Sheffield, Sheffield, UK; 2 School of Health and Related Research, University of Sheffield, Sheffield, UK

**Keywords:** Outcomes research, Asthma, Chronic obstructive pulmonary disease

## Abstract

Substance misuse is associated with poor asthma outcome and death. People with opioid use disorder (OUD) may be at particular risk, however, there have been no case-control studies of asthma care and outcomes in this patient group. A primary care database study of patients with asthma aged 16–65 years was conducted using a matched case-control methodology. The dataset comprised 275,151 adults with asthma, of whom 459 had a clinical code indicating a lifetime history of OUD. Cases with a history of OUD were matched to controls 1:3 by age, gender, smoking status and deprivation index decile. Attendance at annual review (30%) and for immunisation (25%) was poor amongst the overall matched study population (*N* = 1832). Compared to matched controls, cases were less likely to have attended for asthma review during the previous 12 months (OR = 0.60, 95% CI 0.45–0.80) but had similar immunisation rates. Higher rates of ICS (OR = 1.50, 1.13–1.98) and oral prednisolone use (OR = 1.71, 1.25–2.40) were seen amongst those with a history of OUD and 7.2% had a concurrent diagnosis of COPD (OR = 1.86, 1.12–2.40). We found that people with asthma and a history of OUD have worse outcomes on several commonly measured metrics of asthma care. Further research is required to identify reasons for these findings, the most effective strategies to help this vulnerable group access basic asthma care, and to better understand long-term respiratory outcomes.

## Introduction

Asthma is amongst the most common long-term health conditions in the UK^1^ and represents a significant burden for patients, general practice, emergency medicine and secondary care. Small scale observational studies suggest that people with asthma and opioid use disorder (OUD) experience high rates of acute exacerbations^2^, have more severe symptoms during acute episodes^3,4^ and are more likely to need intensive care treatment and intubation^5,6^. The UK has one of the highest rates of death from asthma in Europe^7^ and in the 2014 UK National Review of Asthma Deaths, substance misuse was identified as a factor in 6% of the cases^8^. Supported self-management such as medication adherence^9^, attendance at asthma review^10,11^ and immunisation^12^ are known to improve outcome. However, people with substance use disorders (SUD) often live with significant psychosocial challenges, for example poverty, stigma, family breakdown, insecure housing and incarceration, which may affect their access to healthcare services^13^.

Lack of healthcare utilisation may explain why outcomes amongst people with asthma and SUD appear to be so poor, but the nature of specific illicit drug use might also be a significant additional factor for those with OUD. As a part of the harm minimisation paradigm, injecting heroin users were encouraged to adopt alternative routes of drug use and by the late 1990s, inhalation became a common method of administration in many countries^14,15^. These ‘route transition interventions’ were aimed at reducing the risk of fatal overdose, blood-borne virus transmission and intravenous drug injection-associated infections. However, inhalation of heroin has bronchoconstrictive effects in the lungs^3^, probably mediated by the ability of morphine to cause histamine release^16^, and appears to contribute to progressive irreversible lung disease, such as emphysema and chronic obstructive pulmonary disease (COPD)^17,18^. Assessing the contribution of inhalation of opioids to lung outcomes is challenging as the vast majority of those with SUD smoke tobacco^19^. It is known that people with SUD have significantly higher excess mortality than the general population from causes, including cancer, cardiovascular and respiratory disease^20^, and it is likely that a significant proportion of this excess mortality is explained by tobacco smoking.

At present, there is little existing research that has attempted to quantify asthma outcomes among people with OUD. The majority of existing research comes from small scale and case-series data^17^. More generally, it is recognised that the literature on the pulmonary consequences of heroin use is scant^18^. When assessing asthma outcomes amongst people with OUD, it is important to acknowledge this group are more likely to be male, come from areas of high deprivation and have smoking histories^5^. Factors such as these have been shown to influence asthma outcomes including exacerbation rates^21–24^. This study, aims to assess asthma care and outcomes amongst asthma patients with a history of OUD in a large primary care population after controlling for such confounding.

## Results

### Rates of OUD

**Fig. 1:**
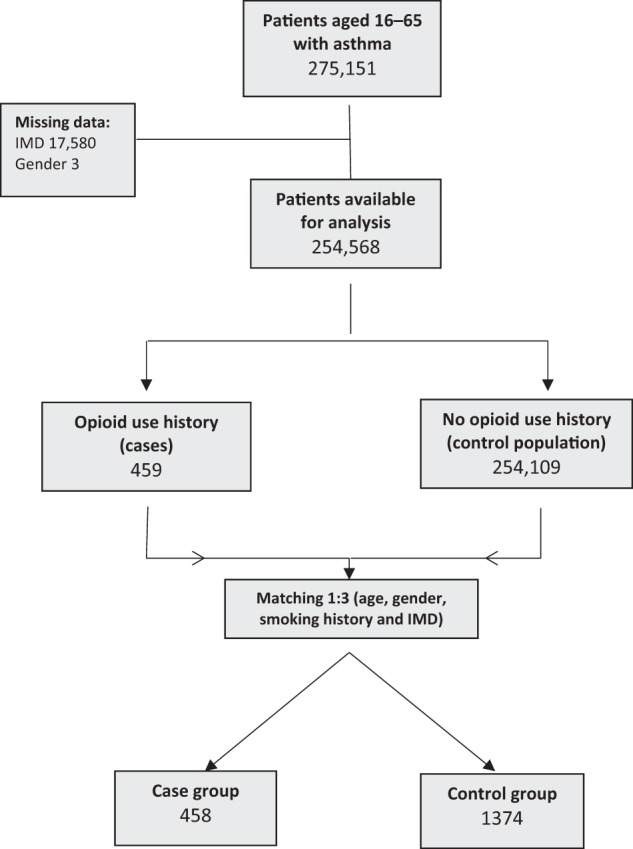
Matched case-control methodology. Flow diagram showing process by which case and control groups were derived from the dataset. IMD index of multiple deprivation.

In all, 275,151 patients aged 16–65 years with asthma were identified from the ResearchOne database. Of these, 459 had a clinical code indicating past or current OUD. Of the total number of patients, 254,568 had complete data available for matching (Fig. 1). There was no difference in the frequency of historical OUD between those with complete and those with missing data (Pearson *χ*^2^ = 1.346, *p* = 0.246).

### Study population characteristics—matched groups

Comparison between the groups with respect to demographics and smoking history are shown in Table 1 along with the results of significance tests. As expected, statistically significant differences with respect to the measured confounding factors were seen. Whilst asthma patients with a history of OUD were only slightly older, they were much more likely to be male and considerably more likely to live in an area with the highest level of deprivation. Ninety five percent of those with a history of OUD had a history of smoking compared to 52% of non-opioid users.

**Table 1:** Study population characteristics.

	History of opioid use	No history of opioid use	Significance tests
Total (%)	459 (0.18%)	254,109 (99.82%)	
Mean age (SD)	39.9 years (8.4)	38.4 years (13.8)	Mean difference (95% CI) 1.53 years (0.27–2.8), *p* = 0.018^a^
% Female	38.8	51.3	*χ*^2^ = 28.16, *p* < 0.001
% Smoking history	94.8	52.0	*χ*^2^ = 335.83, *p* < 0.001
% in most deprived IMD centile	37.5	10.9	*χ*^2^ = 330.50, *p* < 0.001

^a^Independent groups *t*-test, two-tailed *p* value.

### Outcomes—descriptive statistics

After matching, the analysis population comprised 458 asthma patients with a history of past or current OUD (cases) and 1374 without (controls). Table 2 gives values and percentages for each of the outcome variables. Less than one third of the study sample had attended for an asthma review in the previous 12 months, with those in the control group more likely to have attended than cases (31% vs. 26%). More than three quarters of all patients had not received an influenza vaccination during the observation period with marginally higher rates of uptake amongst the control group (25% vs. 23%). Asthma patients from the control group were less likely to have been issued three or more corticosteroid containing inhalers (29% vs. 36%) in the previous 12 months with overall rates for both groups of 31%. Approximately 8% of the 1832 patients received a prescription for oral prednisolone on at least one occasion in the 12-month period with cases recorded as having double the rates of controls (12 vs. 6%). The overall prevalence of COPD amongst the study group was 4.5% with higher rates recorded for those with a history of OUD compared to controls (7.2% vs. 3.6%).

**Table 2:** Outcome variables—study population (*N* = 1832).

Outcome (past 12 months)	History of opioid use (cases) *n* = 458	No history of opioid use (controls) *n* = 1374	All *N* = 1832
	Number %	Number %	Number %
Attended for asthma review	118	427	545
	25.8%	31.1%	29.7%
Received influenza vaccination	105	345	450
	22.9%	25.1%	24.6%
≥3 issued prescriptions for an ICS inhaler	161	402	565
	35.6%	29.3%	30.8%
Issued prescription for oral prednisolone	57	84	141
	12.4%	6.1%	7.7%
Existing clinical code for COPD	33	50	83
	7.2%	3.6%	4.5%

### Outcomes—odds ratios estimated from conditional logistic regression

Crude and adjusted odds ratios (ORs) with 95% confidence intervals (CIs) for all outcome variables are shown in Table 3. Statistically significant ORs were found for all outcomes other than receiving an influenza vaccination (95% CI 0.68–1.14). The adjusted odds of attending for an asthma review were around 40% lower for cases than controls (95% CI 0.45–0.80) whereas the adjusted odds of receiving three or more ICS inhalers (95% CI 1.13 to 2.98), being issued a prescription for oral prednisolone (95% CI 1.25–2.34), or having a pre-existing diagnosis of COPD (95% CI 1.12–3.01) were increased by 50%, 71% and 86% respectively. Crude and adjusted odds ratios were similar suggesting that there were no significant interactions between the outcome variables other than for co-existing COPD in which the adjusted OR fell from 2.26 to 1.86.

**Table 3:** Crude and adjusted odds ratios and 95% CIs from conditional logistic regression analysis.

Outcome (previous 12 months)	Crude OR (95% CI)	*P*-value^a^	Adjusted^b^ OR (95% CI)	*P*-value^a^
Attended for asthma review	0.756 (0.591–0.968)	0.026	0.600 (0.449–0.801)	0.001
Received influenza immunisation	0.878 (0.677–1.139)	0.328	0.796 (0.594–1.067)	0.127
≥3 issued prescriptions for an ICS inhaler	1.357 (1.077–1.708)	0.009	1.497 (1.131–1.982)	0.005
Issued prescription for oral prednisolone	1.779 (1.337–2.367)	<0.001	1.708 (1.247–2.339)	0.001
Existing clinical code for COPD	2.226 (1.371–3.613)	0.001	1.863 (1.120–3.098)	0.017

*OR* odds ratio, *ICS* inhaled corticosteroid, *COPD* chronic obstructive pulmonary disease.

^a^Wald test.

^b^Adjusted for all other outcomes variables in column 1.

## Discussion

Our findings confirm that asthma patients with evidence of previous OUD are more likely to be older, male, come from areas of higher deprivation and have a smoking history. Ignoring, for a moment the role of OUD, we found low rates of access to asthma care and inhaled ICS prescriptions amongst both cases and controls. In matched analyses, opioid users were less likely to attend for annual review but had higher rates of ICS prescriptions. Asthma patients with a history of OUD use had higher rates of oral prednisolone use within the previous 12 months and were more likely to have a co-existing clinical code for COPD. The latter was the only variable, which appeared to be influenced by other outcomes in multi-variable analyses.

Supported self-management forms the cornerstone of asthma treatment and has been shown to reduce hospital admission and unscheduled clinical contacts^25^. It was therefore, striking to find such low rates of achievement of the quality indicators for asthma care in this study. Less than one third of the 1832 patients had attended for an asthma review in the past 12 months and less than a quarter had received an influenza vaccination. This is despite these aspects of asthma care being incentivised in England through the Quality Outcomes Framework (QoF) and the Enhanced Services Specification^26^, respectively. The rates of attendance an asthma review in our matched sample were considerably lower than the rates among the UK general population of asthma patients in similar database. For example, in a recent study using the Clinical Practice Research Datalink (CPRD) (an alternative UK GP database to ResearchOne), 43.7% of patients had attended for asthma review^27^. After controlling for measured confounding and other outcomes variables, we found that OUD was associated with a 40% reduction in the odds of having attended for asthma review. The finding of higher rates of ICS prescriptions among those with a history of OUD is somewhat at odds with this and has several possible interpretations. Assuming that both groups have similar severity of asthma requiring regular ICS-preventer therapy, then, taken at face value, this suggests higher adherence amongst those with a history of OUD. In the context of other chronic health conditions such as HIV, medication adherence is known to be lower in those with SUD^28^ and so this would seem unlikely. An alternative explanation is that clinical need for an ICS-containing inhaler was different between the two groups and the higher OR is a reflection of this rather than differences in adherence per se. This would be consistent with the notion that OUD is associated with poorer lung function in those with asthma. It is also known that use of ICS increases during the period before and after exacerbations^29^, which could also explain the direction of this outcome. A national sample of data from the CPRD reported by Bloom et al., indicate that around 65% of people with asthma aged 18–54 years received ≥3 ICS inhalers in 2016^27^. Therefore, irrespective of the interpretation of the differences between those with a history of OUD and their matched counterparts, the rates of ICS prescriptions seen in this study as well as attendance at asthma review are worryingly low. In a systematic review of strategies for implementing supported self-management for people with asthma^11^, Pinnock and colleagues found that that a whole system approach, targeting patients, professionals and organisations was crucial to improving global asthma care outcomes and that effective interventions were characterised by multi-faceted proactive care. These authors also call for further research into how whole system approaches can be integrated into the routine care of people with asthma and comorbidities as well demographically diverse groups^11^.

There is consensus that the definition of asthma exacerbation should include the systemic use of oral corticosteroids (OCS)^30^ and prescriptions for oral prednisolone have previously been used as a measure of exacerbation from database studies^27,31^. The present study findings, suggest that OUD is associated with increased rates of asthma exacerbations. In addition to the acute risks, asthma exacerbations are independent predictors of future exacerbation^32^ and are correlated with the irreversible airflow limitations over time^33^. There is increasing evidence that inhalation of opioids results in airways disease and in a recent screening study, Burhan et al.^34^ found that most heroin smokers screened in a community setting showed evidence of spirometry-confirmed COPD or COPD-asthma overlap. The present study complements this work as it demonstrates that asthma patients with a history of opioid use have greater rates of COPD independent of some of the main risk factors for the development of this condition, namely tobacco smoking history, deprivation and age^35^. Concurrent diagnoses of asthma and COPD are associated with higher all-cause mortality^36^ and our results suggest that asthma patients with a history of OUD are almost twice as likely to have a comorbid diagnosis of COPD. The odds ratio in our multivariable (adjusted) analysis for the presence of a COPD diagnosis was lower than in the univariate analysis. One tentative interpretation of this is that reducing exacerbations and/or improving engagement with self-supported care could influence the development of COPD. Further research could explore this possibility by replicating this work in alternative primary care databases.

Clinical recording systems such as ResearchOne and similar databases are widely used and validated for asthma epidemiological research^37^. However, it is known that substance use is under-recorded in primary care databases^38^. In the present study, only 0.2% of those with a history of asthma were identified as also having a clinical code associated with OUD. The decisions taken by primary care physicians to record substance use are complex^39^ and therefore it is necessary to be cautious in generalising the present results. As this was a database study, it was also not possible to determine current OUD and the findings therefore relate to a lifetime history which may include both current and past OUD. We chose not to omit those who did not have evidence of active asthma, such as prescription issues within an arbitrary timeframe and did not examine exception reporting (a process by which a patient may be ‘excepted’ from the targets set in a particular clinical area of the QOF on certain grounds or informed dissent). This was done in an attempt to avoid a potential source of selection bias. There are several potential undesirable consequences of this. Firstly, it is possible that the overall low rates of measured outcomes were simply due to some patients having resolved asthma but with their Read Code not appropriately removed. Secondly, patients may have poorer engagement in part because they have been exception reported. This could potentially explain some of the differences seen between the cases and controls from this study. With respect to ICS use, although our method was pre-specified, a more formal measure of adherence such as the Annual Prescription Possession method may have been desirable to aid comparison with existing literature. Some qualification is also required with respect to matching. As only a basic measure of tobacco smoking history was used (absence or presence) it possible that those with a history of OUD were heavier smokers than those without such histories. More generally, as this is an observational study, there may have been detrimental exposures in the OUD group which were not measured.

Taken together, the results of this study suggest that people with OUD have poor access to asthma care/self-management and worse outcomes on several important measures, despite controlling for important factors which are known to have deleterious effects on asthma, such as tobacco smoking and lower socioeconomic status. Those with a history of OUD encounter a complex range of negative determinants of health at a patient, provider and health system level in common with patients from other populations such as those with severe mental health problems. Further research is required to identify reasons for poor asthma care amongst those from vulnerable and marginalized populations at a whole system level and to better understand how OUD affects long-term respiratory outcomes.

## Methods

### Data source

Data came from the ResearchOne clinical research database^40^, which contains pseudo-anonymised pooled data from participating General Practices in England, UK which use the System One clinical recording system. The database contains information from the patient’s electronic health record including coded clinical entries called Read codes (the hierarchical clinical terminology system used by UK General Practices^41^) and prescribing data. Research ethics approval was granted from North West—Greater Manchester East Research Ethics committee on 12 January 2016 (16/NW/0037). Data were requested in the form of Read codes and British National Formulary (BNF) chapter heading codes. The observation period was the 12 months prior to the 8 October 2016.

### Study design, population and outcome measures

A matched case-control study was conducted to examine differences between asthma patients with and without a history of OUD. The study population consisted of patients in ResearchOne-linked practices with specific Read codes indicating a possible diagnosis of asthma. A Read code list for this purpose (shown in Supplementary Table 1) was compiled following consultation with a public health specialist. This method has previously been shown to have a high positive predictive value for the diagnosis of asthma^42^. Patients were eligible for inclusion if they were aged 16–65 years. Individuals with a history of OUD (cases) were identified using the Read code list shown in Supplementary Table 2. As it is not possible to ascertain current vs. past OUD (or OUD in remission), ‘history’ here refers to lifetime history of OUD.

Variables of interest were selected to assess important aspects of asthma care and outcomes. Asthma care measures were based on the British Thoracic Society and Scottish Intercollegiate Guidelines Network (BTS/SIGN) British Guidelines on the management of asthma^43^. These were: attendance at annual asthma review; receiving an influenza vaccination and being issued three or more prescriptions for a corticosteroid (ICS)-containing inhaler as preventer therapy. The cut-off of three ICS-containing inhalers was selected as providing the minimum number of inhalers in order to achieve an approximate 80% adherence for a typical 200-metered dose inhaler at two puffs per day. Annual asthma reviews, in the UK, are structured consultations between a patient and, usually, a nurse, with the aim of assessing current control of symptoms and important aspects of self-management. These are recommended in the UK national guidelines and are a NICE Quality and Outcomes Framework indicator^1^.

In addition, as a proxy measure of acute asthma exacerbation, we examined whether any prescriptions of oral prednisolone had been issued over the 12-month period. In order to minimise the chances of selecting prednisolone prescribed for alternative indications such as rheumatological conditions, we selected only 5 mg tablet issues with quantities which would correspond to a 5 to 7-day course of 30–40 mg. There is increasing recognition of overlap between asthma and chronic obstructive pulmonary disease (COPD)^44^. Progression of asthma to COPD may be driven, in part, by exacerbations^45^ and there is evidence of an association between heroin inhalation and the early development of emphysema^46^. We, therefore, sought to explore this further by including comorbid diagnoses of COPD. Comorbid diagnoses of COPD were identified through the selection of appropriate Read codes shown in Supplementary Table 3.

### Matching

Cases and controls were matched 1:3 on four confounding variables: age (within 3 years), gender, any recorded smoking history and socioeconomic status (SES). Multiple controls allow for increased statistical power and whilst there is a general consensus that increasing the case-to-control ratio above 1:4 results in very little gain in efficiency, some authors have argued for greater ratios under certain circumstances^47^. The trade-off for this however is that there may be a reduction in the overall matched sample size. In the present study, a 1:3 ratio resulted in the loss of only one case and was therefore chosen as the best compromise between statistical efficiency and matched sample size. The English 2010 Index of Multiple Deprivation (IMD) was used as the SES measure for this study. The IMD contains 38 indicators, organised across seven domains of deprivation which are combined to calculate an overall score representing the degree of deprivation experienced by people living in an area^48^. The IMD score associated with the patient’s last known address during the observation period was used and converted to deciles for the purpose of matching.

### Statistical analyses

Differences between asthma patients with and without a history of OUD with respect to the matching variables were made for illustrative purposes. Proportions were assessed using chi-squared tests and mean differences in age were assessed with 95% confidence intervals and a two-tailed independent group’s t-test. Adjusted and unadjusted (crude) odds ratios and 95% confidence intervals were calculated using conditional logistic regression^49^. All analyses and data handling were conducted using SPSS version 24. Matching was conducted using the SPSS Python Essentials case-control matching module.

### Reporting summary

Further information on research design is available in the Nature Research Reporting Summary linked to this article.

## Supplementary information

Supplementary Information

Reporting Summary

## Data Availability

The data that support the findings of this study are available from ResearchOne but restrictions apply to the availability of these data, which were used under license for the current study, and so are not publicly available. Data are, however, available from the authors upon reasonable request and with permission of ResearchOne. The approach taken for the study is detailed in the main text and, and could be reproduced in any similarly structured linked database.

## Code availability

No custom software was developed. Only standard use of existing routines within software for data manipulation and statistical analysis was employed.
