# Supplementary material for: A primary care database study of asthma among patients with and without opioid use disorders
Source: NPJ Prim Care Respir Med. 2020 Apr 20;30:17. doi: 10.1038/s41533-020-0174-2 (PMC7170905; doi:10.1038/s41533-020-0174-2)
Supplement: Supplementary file 1 — Supplementary Information [file 41533_2020_174_MOESM1_ESM.pdf]

Supplementary material

Supplementary Table 1. Read codes used to identify patients with a diagnosis of asthma

Description	Code
Exercise-induced asthma	173A.
Chronic asthmatic bronchitis	H3120
Asthma	H33..
Asthma: [extrins - atop][allerg][pollen][childh][+ hay fev]	H330.
(Hay fever + asthma) or (extr asthma without status asthmat)	H3300
Extrins asthma with: [asthma attack] or [status asthmaticus]	H3301
Extrinsic asthma NOS	H330z
(Intrinsic asthma) or (late onset asthma)	H331.
Intrinsic asthma without status asthmaticus	H3310
Intrins asthma with: [asthma attack] or [status asthmaticus]	H3311
Intrinsic asthma NOS	H331z
Mixed asthma	H332.
Asthma unspecified	H33z.
(Severe asthma attack) or (status asthmaticus NOS)	H33z0
Asthma attack (& NOS)	H33z1
Late onset asthma	H33z2
(Asthma:[exerc ind][allerg NEC][NOS]) or (allerg bronch NEC)	H33zz
Brittle asthma	Ua1AX
Childhood asthma	X101t
Late onset asthma	X101u
Allergic asthma	X101x
Extrinsic asthma with asthma attack	X101y
Allergic asthma NEC	X101z
Hay fever with asthma	X1020
Allergic non-atopic asthma	X1021
Intrinsic asthma with asthma attack	X1022
Aspirin-sensitive asthma with nasal polyps	X1024
Status asthmaticus	X102D
Asthmatic bronchitis	Xa0lZ
Acute asthma	Xa9zf
Chronic asthma with fixed airflow obstruction	Xaa7B
Asthma-chronic obstructive pulmonary disease overlap syndrom	Xac33
Nocturnal asthma	XaLPE
Allergic atopic asthma	XE0YQ
Extrinsic asthma without status asthmaticus	XE0YR

Supplementary Table 1. Read codes used to identify patients with a diagnosis of asthma (continued)

Description	Code
Extrinsic asthma with status asthmaticus	XE0YS
Non-allergic asthma	XE0YT
Intrinsic asthma with status asthmaticus	XE0YU
Status asthmaticus NOS	XE0YV
Asthma attack	XE0YW
Asthma NOS	XE0YX
Extrinsic asthma - atopy (& pollen)	XE0ZP
Asthma: [intrinsic] or [late onset]	XE0ZR
Asthma: [NOS] or [attack]	XE0ZT
Asthma attack NOS	XM0s2

Supplementary Table 2. Read codes used to identify patients with a history of opioid use disorder (OUD)

Description	Code
Heroin dependence	X00Rz
Drug addiction therapy - methadone	XaCMF
Heroin misuse	XaLQN
Methadone maintenance therapy	Ua1MY
History of heroin misuse	XaMgO
Previous history of opiate misuse	XaMfk
History of opiate misuse	XaMfE
Previous history of heroin misuse	XaMfL
Methadone Reduction Therapy	Y0db3
History of daily heroin misuse	XaMfJ
Uses heroin on top of substitution therapy	XaMhO
Combination of crack and heroin	Xa47H
History of daily opiate misuse	XaMfh
Nondependent opioid abuse NOS	E255z
Chases the dragon	Ub0n3
History of methadone misuse	XaMf3
Nondependent opioid abuse	E255.
History of infrequent heroin misuse	XaMfM
Previous history of methadone misuse	XaMfQ
History of buprenorphine misuse	XaXHJ
High dose methadone replacement	Y0a42
Average amount of opiates on a using day	Y07c4
Heroin - non-pharmaceutical	Ub18B

Supplementary Table 3. Read codes used to identify patients with a history of COPD

Description	Code
Chronic obstructive lung disease	H3...
Chronic bronchitis	H31..
Simple chronic bronchitis	H310.
Simple chronic bronchitis	H3100
Simple chronic bronchitis NOS	H310z
Mucopurulent chronic bronchitis	H311.
Purulent chronic bronchitis	H3110
Mucopurulent chronic bronchitis NOS	H311z
Chronic asthmatic bronchitis	H3120
Emphysematous bronchitis	H3121
Obstructive chronic bronchitis NOS	H312z
Mixed simple and mucopurulent chronic bronchitis	H313.
Other chronic bronchitis	H31y.
Chronic tracheobronchitis	H31y1
Other chronic bronchitis NOS	H31yz
Chronic bronchitis NOS	H31z.
Emphysema	H32..
Chronic bullous emphysema	H320.
Segmental bullous emphysema	H3200
Zonal bullous emphysema	H3201
Giant bullous emphysema	H3202
Chronic bullous emphysema NOS	H320z
Panlobular emphysema	H321.
Centrilobular emphysema	H322.
Other emphysema	H32y.
Acute vesicular emphysema	H32y0
MacLeods syndrome	H32y2
(Sawyer-Jones syndrome) or (other emphysema NOS)	H32yz
Emphysema NOS	H32z.
Other specified chronic obstructive airways disease	H3y..
Chronic obstructive airways disease NOS	H3z..
Chronic emphysema due to chemical fumes	H4640
Toxic bronchiolitis obliterans	H4641
[X]Other emphysema	Hyu30
[X]Other specified chronic obstructive pulmonary disease	Hyu31
Occupational chronic bronchitis	X101j
Byssinosis grade 3	X101k
Bronchiolitis obliterans	X101l
Drug-induced bronchiolitis obliterans	X101m
Pulmonary emphysema	X101n

Supplementary Table 3. Read codes used to identify patients with a history of COPD (continued)

Description	Code
Pulmonary emphysema in alpha-1 PI deficiency	X101o
Toxic emphysema	X101p
Congenital lobar emphysema	X101q
Scar emphysema	X101r
Bronchiolitis obliterans with usual interstitial pneumonitis	X102z
Eosinophilic bronchitis	Xaa7C
Asthma-chronic obstructive pulmonary disease overlap syndrom	Xac33
Mild chronic obstructive pulmonary disease	XaEIV
Moderate chronic obstructive pulmonary disease	XaEIW
Severe chronic obstructive pulmonary disease	XaEIY
End stage chronic obstructive airways disease	XaIND
Very severe chronic obstructive pulmonary disease	XaN4a
Purulent chronic bronchitis	XE0YM
Bullous emphysema with collapse	XE0YN
Atrophic (senile) emphysema	XE0YO
Other emphysema NOS	XE0YP
Chronic: [bronchitis NOS] or [tracheobronchitis]	XE0ZN
